# Formaldehyde treatment of proteins enhances proteolytic degradation by the endo-lysosomal protease cathepsin S

**DOI:** 10.1038/s41598-020-68248-z

**Published:** 2020-07-14

**Authors:** Thomas J. M. Michiels, Hugo D. Meiring, Wim Jiskoot, Gideon F. A. Kersten, Bernard Metz

**Affiliations:** 1 Division of BioTherapeutics, Leiden Academic Centre for Drug Research (LACDR), Leiden University, 2333 CC Leiden, The Netherlands; 2 Intravacc, Institute for Translational Vaccinology, 3721 MA Bilthoven, The Netherlands

**Keywords:** Chemical modification, Lysosomes

## Abstract

Enzymatic degradation of protein antigens by endo-lysosomal proteases in antigen-presenting cells is crucial for achieving cellular immunity. Structural changes caused by vaccine production process steps, such as formaldehyde inactivation, could affect the sensitivity of the antigen to lysosomal proteases. The aim of this study was to assess the effect of the formaldehyde detoxification process on the enzymatic proteolysis of antigens by studying model proteins. Bovine serum albumin, β-lactoglobulin A and cytochrome c were treated with various concentrations of isotopically labelled formaldehyde and glycine, and subjected to proteolytic digestion by cathepsin S, an important endo-lysosomal endoprotease. Degradation products were analysed by mass spectrometry and size exclusion chromatography. The most abundant modification sites were identified by their characteristic MS doublets. Unexpectedly, all studied proteins showed faster proteolytic degradation upon treatment with higher formaldehyde concentrations. This effect was observed both in the absence and presence of glycine, an often-used excipient during inactivation to prevent intermolecular crosslinking. Overall, subjecting proteins to formaldehyde or formaldehyde/glycine treatment results in changes in proteolysis rates, leading to an enhanced degradation speed. This accelerated degradation could have consequences for the immunogenicity and the efficacy of vaccine products containing formaldehyde-inactivated antigens.

## Introduction

Enzymatic degradation of antigens is a crucial step in the process of acquiring cellular immunity, e.g., through the induction of antigen specific T-helper cells or cytotoxic T-cells. The endo-lysosomal protease activity is lower for immune cells with high antigen presentation capacity, such as dendritic cells, than for cells with lower antigen presentation capacity, such as neutrophils.^1^ Several groups have found a correlation between slow proteolytic antigen degradation and increased immunogenicity of the studied antigen,^2-9^ although it does not hold up for all antigens.^10^ This correlation suggests that protease resistance could be an important factor for vaccine efficacy. Currently numerous efforts are being made to replace, reduce and refine (3Rs) the use of animal tests.^11,12^ One approach to achieve this is the so-called consistency approach.^13^ This approach is based on the principle that if a panel of in-vitro tests can prove that a vaccine product is produced in a consistent manner, reduction or replacement of quality control animal tests is possible. An in-vitro test that could follow changes in enzymatic degradation kinetics of protein antigens may thus be used to monitor vaccine batch quality in a consistency approach.

Many antigens in inactivated vaccine products, such as bacterial toxins and poliovirus, are inactivated by using a mixture of formaldehyde and amino acids. This treatment results in modifications to the protein, as reported previously by Metz et al*.* (Table 1.^14,15^ These modifications may alter the immunogenicity of the antigen. For instance, for diphtheria toxoid the immunogenicity increases upon increased exposure to formaldehyde.^16^ Previous research on pertussis antigens indicates a slower in-vitro proteolysis of formaldehyde-treated proteins than untreated counterparts.^17^ However, that study used trypsin as an enzyme, which cleaves after lysine and arginine residues. Both of these amino acids are susceptible to formaldehyde modification, especially the latter being converted in substantial amounts.^15^ Formaldehyde modification of trypsin’s cleavage sites is very likely to affect the substrate’s degradation kinetics, as lysine modifications inhibit digestion by trypsin, but this might be less relevant for lysosomal degradation in antigen-presenting cells (APCs).^18^ Thus, these results might not be indicative of the effect formaldehyde treatment has on the endo-lysosomal degradation kinetics of the antigen.

**Table 1:** Overview of the most common formaldehyde-induced modifications of proteins.

Example structure	Reactive residues	ΔMass (Da)	Conversion rate (%)^a^
	K, Q, N, W, H	+ 12.000	–^b^
	Free *N* termini	+ 12.000	76
	K, Y	+ 12.000	–^b^
	K	+ 14.016	–^b c^
	K, R	+ 24.000	–^b^
	K, Q, N, W, H	+ 30.011	–^b^
	H, W, Q, N	+ 87.032	3.6–6.6
	Y	+ 87.032	62
	R	+ 99.032	56
		+ 198.064^d^	41

^a^Conversions as reported by Metz et al*.*^15^.

^b^Conversion rates not reported.

^c^Product reported by Trezl et al*.*^23^.

^d^Double + 99 modification.

To assess the effect of formaldehyde treatment on enzymatic protein processing three formaldehyde-treated model proteins were prepared: bovine serum albumin (BSA), β-lactoglobulin A and cytochrome c. These proteins were chosen for their structural diversity, commercial availability and their low toxicity (as compared to toxins). These proteins were treated with increasing concentrations of formaldehyde and glycine. They were then subjected to proteolytic digestion by cathepsin S, an important endo-lysosomal enzyme.^19^ The degradation of the intact proteins was monitored by size exclusion chromatography (SEC) and the resulting peptides were identified and quantified using nanoscale liquid chromatography-mass spectrometry (LC–MS).

## Results and discussion

### Enzymatic degradation of formaldehyde-treated model proteins by cathepsin S

**Figure 1:**
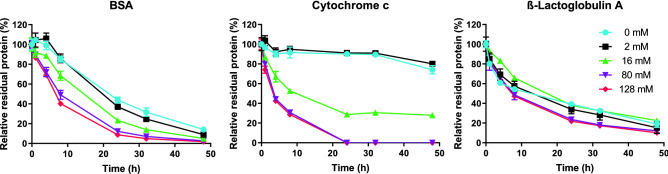
SEC analysis of intact formaldehyde- and glycine-treated proteins as function of cathepsin S digestion time. Error bars represent SD of three enzyme reactions.

Formaldehyde and glycine-treated cytochrome c, BSA and β-lactoglobulin A were subjected to enzymatic degradation by cathepsin S. The digestion was followed by using SEC and LC–MS. The formaldehyde and glycine-treated proteins eluted slightly earlier with SEC than their untreated counterparts. The treated proteins showed broader chromatographic peaks, which were not fully resolved, and a higher dimer content than the untreated proteins (Fig. S1). To allow for more robust quantification, peak height rather than peak area was used, as it was difficult to reliably determine the peak edge of formaldehyde-treated samples at the end of the digestion. Regarding the degradation kinetics, SEC analysis (Fig. 1) showed that the higher the formaldehyde/glycine concentrations the protein was exposed to, the faster the decrease of intact protein content was upon subsequent exposure to cathepsin S. This effect is most clear for cytochrome c and BSA, but is also observed for β-lactoglobulin.

**Figure 2:**
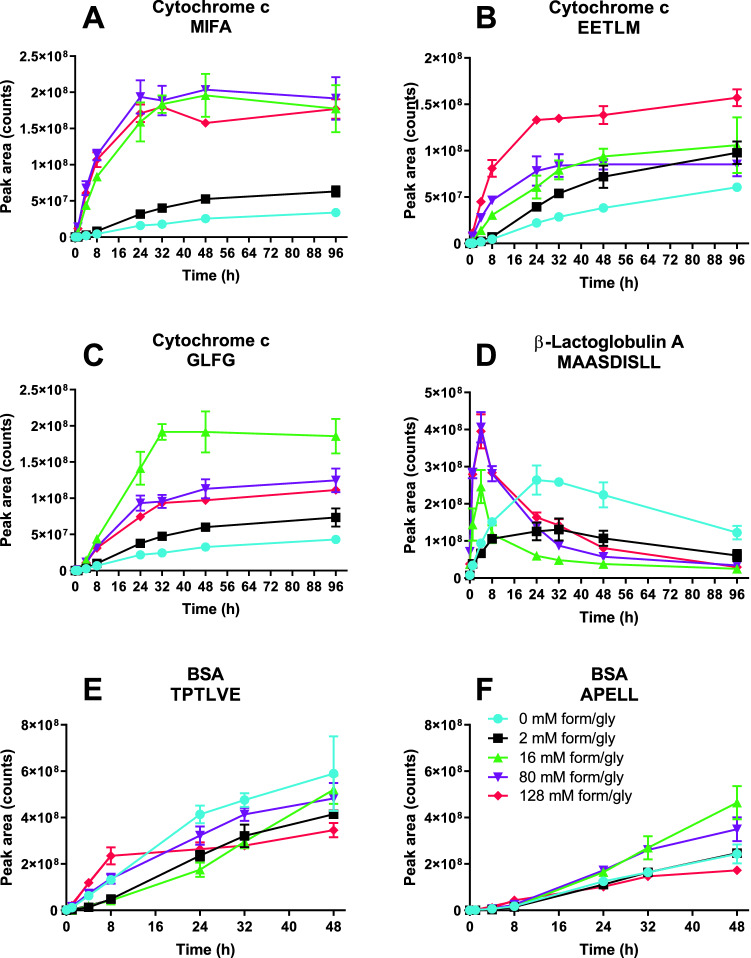
Illustrative examples of LC–MS analysis of degradation products that do not contain formaldehyde reactive amino acids and are derived from formaldehyde- and glycine-treated proteins, as function of cathepsin S digestion time. Error bars represent SD of three enzyme reactions. (**A**) Kinetics of the cytochrome c peptide MIFA. (**B**) Kinetics of the cytochrome c peptide EETLM. (**C**) Kinetics of the cytochrome c peptide GLFG. (**D**) Kinetics of the β-lactoglobulin A peptide MAASDISLL. (**E**) Kinetics of the BSA peptide TPTLVE. (**F**) Kinetics of the BSA peptide APELL.

Peptides that are sensitive to formaldehyde-induced modifications would not allow for direct comparison between samples, because the ratio modified/unmodified peptide depends on the formaldehyde/glycine concentrations. Furthermore, variety in MS response factors between the modified and unmodified peptides would have to be taken into account, as the proton affinity of an unmodified side chain would likely be different to that of a modified side chain.^20^ Other difficulties with direct comparison arise from cross-linked dipeptides, which cannot be identified automatically by the PEAKS software (or equivalent software packages), and from the fact that cross-linked dipeptides and adducts give challenging Collision Induced Dissociation (CID) fragmentation spectra with neutral losses. The latter problem results in exclusion based on Peptide to Spectrum Match (PSM) score. The challenging neutral losses from CID fragmentation can often be avoided by using Electron Transfer Dissociation (ETD) fragmentation, but this requires multiply charged (z > 2) species at relatively high abundance.^21^ To overcome these limitations and avoid bias, only the formation kinetics of some of the most abundant peptides that are considered inert, *i.e.*, not modifiable by formaldehyde, are plotted in Fig. 2 15. These representative peptides were selected to include peptides with various kinetic profiles. Length variants with the same core sequence were excluded.

LC–MS analysis showed that inert peptides were formed faster after exposure to formaldehyde and glycine. For instance, the cytochrome c peptide MIFA reached a maximum degradation speed upon treatment with 16 mM formaldehyde/glycine (Fig. 2A); higher formaldehyde/glycine concentrations did not alter the kinetics further. Another cytochrome c peptide, EETLM, showed a more gradual increase in formation rate with increasing formaldehyde/glycine concentrations (Fig. 2B). Cytochrome c peptide GLFG showed an optimum degradation speed at 16 mM formaldehyde/glycine (Fig. 2C), although even exposure to the highest concentrations still enhanced the formation of this peptide compared to the control (i.e., cathepsin S-treated cytochrome c that was not previously exposed to formaldehyde/glycine). The β-lactoglobulin peptide MAASDISLL is an example of an intermediate product that likely was degraded to smaller peptides by the enzyme. This peptide was formed faster with higher formaldehyde/glycine concentrations, with the peptide’s maximum intensity shifting to earlier time points (Fig. 2D). BSA showed relatively few unreactive peptides compared to the smaller cytochrome c and ß-lactoglobulin. This is mainly due to the high number of cysteines (35 per BSA molecule) on top of the other reactive amino acids. The usual reduction/alkylation was not performed to avoid unwanted side reactions (with the formaldehyde modifications) and protein unfolding. While cystines are not reactive towards formaldehyde,^15^ the cysteines formed under the reaction conditions impede quantification of the peptides that contain them. Peptides TPTLVE and APELL (Fig. 2E,F) were characteristic for the BSA peptides. TPTLVE showed a decrease in formation speed compared to the control when the formaldehyde and glycine concentration was low (2 and 16 mM), but higher concentrations (80 mM and 128 mM) showed a recovery of the original degradation speed. The APELL degradation profile is somewhat similar to cytochrome c’s GLFG kinetics, with an optimum at intermediate concentrations (16 and 80 mM), although the differences in degradation rate between the treatments are small.

### Role of glycine in the increased enzymatic degradation upon formaldehyde/glycine treatment

From the above, it cannot be concluded to what extent the presence of glycine contributes to the observed increase in enzymatic degradation. Therefore, we performed a follow-up experiment in which the most susceptible protein, cytochrome c, was treated with only formaldehyde (128 mM, **CYT-F128-1**) or with formaldehyde (128 mM) and glycine (128 mM, **CYT-FG128-1**) for one week, to evaluate whether formaldehyde-glycine adducts affect the increased proteolytic degradation rate.

**Figure 3:**
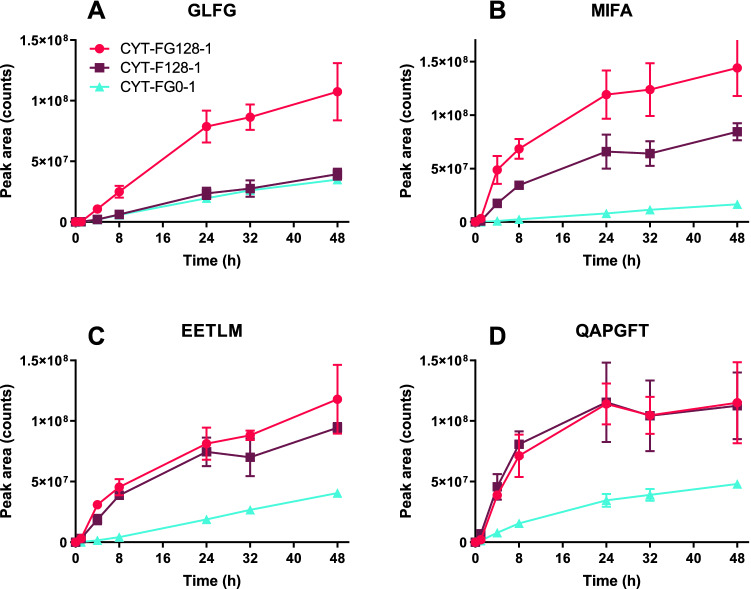
Effect of the presence of glycine during formaldehyde treatment on the subsequent enzymatic degradation rate of cytochrome c upon digestion by cathepsin S. Peptide formation was quantified by using LC–MS for samples/conditions **CYT-FG0-1**, **CYT-F128-1** and **CYT-FG128-1**. Peptide peak areas are corrected for the internal standard peak area. Error bars represent the SD of three LC–MS runs. (**A**) Kinetics of the cytochrome c peptide GLFG. (**B**) Kinetics of the cytochrome c peptide MIFA. (**C**) Kinetics of the cytochrome c peptide EETLM. (**D**) Kinetics of the cytochrome c peptide QAPGFT.

Reaction **CYT-FG128-1** resulted in an increase in the formation of the GLFG peptide compared to both control reaction **CYT-FG0-1** and reaction **CYT-F128-1 (**Fig. 3A). This indicates involvement of one or more formaldehyde-glycine adducts in the proteolytic process leading to the formation of GLFG. For the peptides EETLM and QAPGFT, however, **CYT-F128-1** and **CYT-FG128-1** resulted in a similar kinetic profile. So, modifications caused by formaldehyde itself were responsible for the increase in proteolytic degradation rate (Fig. 3C, D). For the MIFA peptide, both treatments showed a large increase in peptide formation rate compared to the control sample, although the formaldehyde-/glycine-treated sample showed a larger effect than the sample treated with only formaldehyde (Fig. 3B). Altogether, these results indicate that, depending on the proteolytic cleavage site, the formation of formaldehyde adducts and/or crosslinks and formaldehyde-glycine adducts contribute to the altered proteolytic peptide formation kinetics.

### Location of the most abundant modifications and their solvent accessibility

**Figure 4:**
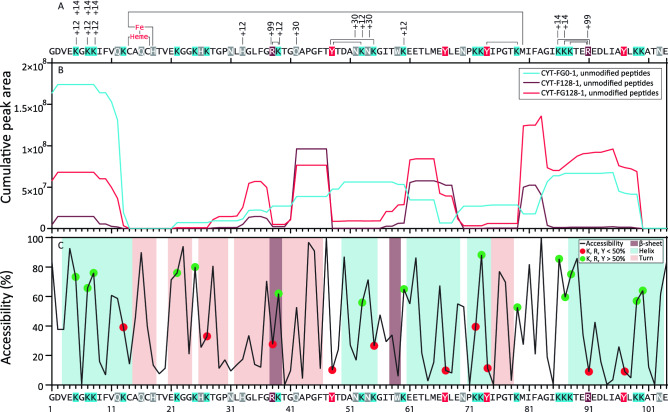
Graphical representation of formaldehyde-induced modifications and enzymatic degradation within the cytochrome c sequence. (**A**) All identified formaldehyde-induced modifications are annotated in this graph, crosslinks are represented by lines linking the crosslinked residues, imine formation is annotated with + 12, methylation is annotated with + 14, methylol adducts are annotated with + 30, formaldehyde-glycine adducts on arginine residues are annotated with + 99. See Table 1 for chemical structures of the modifications. Histidine 18 and methionine 81 are axial ligands to the haem iron, also indicated by a solid line. Lysine residues are marked blue, tyrosine residues are marked red, arginine residues are marked brown, and other potentially reactive amino acid residues are marked grey. (**B**) Peptide intensity relative to the amino acid sequence. The peptide areas that contained a certain amino acid were summed. Only peptides without formaldehyde modifications were used. The data used was obtained from the 8 h time point of the cathepsin S digestion. Data is plotted for CYT-FG0-1, CYT-F128-1 and CYT-FG128-1. (**C**) Accessibility calculated using GETAREA on 1HRC.^29^ Secondary structures were calculated using STRIDE on 1HRC.^30^ Green dots represent accessible tyrosine, lysine, or arginine residues (> 50%). Red dots represent less accessible tyrosine lysine or arginine residues (< 50%).

By using stable isotope labelling for both formaldehyde (CD_2_O) and glycine (^13^C), doublet peaks in the MS1 spectra of modified peptides formed by cathepsin S digestion were obtained from samples **CYT-F128-1** and **CYT-FG128-1** (Fig. S2). Identification of these doublet peaks allowed us to elucidate the majority of the most abundant amino acid residue modifications and compare these to the residues’ solvent accessibility. In combination with this data, the summed area responses of unmodified peptides originating from the protein can be plotted against the protein’s amino acid sequence to distinguish areas of the protein where the enzymatic degradation is affected by the formaldehyde/glycine treatment (Fig. 4). This plot can then be used to reveal specific modifications responsible for altered enzymatic degradation kinetics. If more peptide is modified, the potential maximum amount of unmodified peptide decreases.

Several regions show clear differences in peptide abundance between the different treatments, which corresponds with differences in the modifications near these regions. For instance, the unmodified *N*-terminal peptides remaining in the formaldehyde-/glycine-treated sample showed a strong decrease in abundance compared to the control sample. This site contains three lysine residues that are susceptible to modification, especially imine formation (Δmass =  + 12 Da (Δm + 12)) and methylation (Δm + 14). Remarkably, R38 was modified both into a formaldehyde-glycine Δm + 99 adduct and into a Δm + 24 lysine crosslink and combinations thereof, despite having a relatively low (ca. 25%) accessibility. The region between Y48 and K60 also showed a low abundance of unmodified peptides for formaldehyde-/glycine-treated protein. For this region, crosslinks between the lysine residues and the tyrosine residue, imine formation on the lysine residues, and methylol formation on the asparagine residue were identified. Another important difference between **CYT-FG0-1** and **CYT-F128-1** and between **CYT-FG0-1** and **CYT-FG128-1** was observed in the region between N70 to K79. Here, the main identified modification was the crosslink between K79 and Y74. The preceding peptide EYL was more abundant in the samples exposed to formaldehyde, therefore this region showed a sharp increase in the overall peptide abundance compared to the control sample, despite Y67 being a potentially reactive residue. No modifications of this residue were identified, implying that the changes in its abundance must originate from modifications elsewhere, possibly the adjacent K79-Y74 crosslink. The unmodified residues near the C-terminus, such as Y97, K99 and K100, had similar profiles for **CYT-FG128-1** compared to **CYT-FG0-1**, with the former being slightly more intense. However, **CYT-F128-1** showed a dramatic decrease in unmodified peptide abundance in this region, indicating major changes compared to the other samples. Modifications on the more buried residues of a protein are more likely to affect protein folding than residues that are more solvent-exposed. Interestingly, the two arginine residues and all tyrosine residues involved in modifications have low solvent accessibility, implicating potential structural changes after modification. Overall, the most striking difference between **CYT-FG0-1** and both **CYT-F128-1** and **CYT-FG128-1** is that exactly the regions with unreactive or unreacted amino acids were far more abundant for **CYT-F128-1** and **CYT-FG128-1**. Metz et al. identified the most common modifications caused by formaldehyde/glycine treatment.^14,15^ The modifications found in cytochrome c correspond to these modifications, with the addition of methylation of lysine residues in an Eschweiler–Clarke reaction.^22^ This reaction has also previously been described for aqueous *N*^α^-Acetyl-l-lysine upon exposure to formaldehyde.^23^

### Modification of R38 results in faster degradation of surrounding sequence

Some modifications that could explain the accelerated formation of the peptide GLFG upon exposure to formaldehyde and glycine were identified. In the cytochrome c sequence, GLFG is directly followed by R38, allowing for formaldehyde-glycine adducts. The peptide RKTGQAPGFT, immediately following GLFG in the cytochrome c sequence, was identified along with various related, modified peptides. The Δm + 99 arginine modification (i.e., 2 formaldehyde groups and 1 glycine group) and the Δm + 111 (+ 99 on R and + 12 on K) modifications were identified. The spectra contained the expected doublet peaks associated with addition of two formaldehyde molecules and one glycine molecule, and three formaldehyde molecules plus one glycine molecule, respectively. Both products showed the characteristic neutral loss of 87.03 Da upon CID fragmentation. The MS^1^ peak intensities of these peaks were similar after 8 h of digestion for all four peptides (Fig. S3).

**Figure 5:**
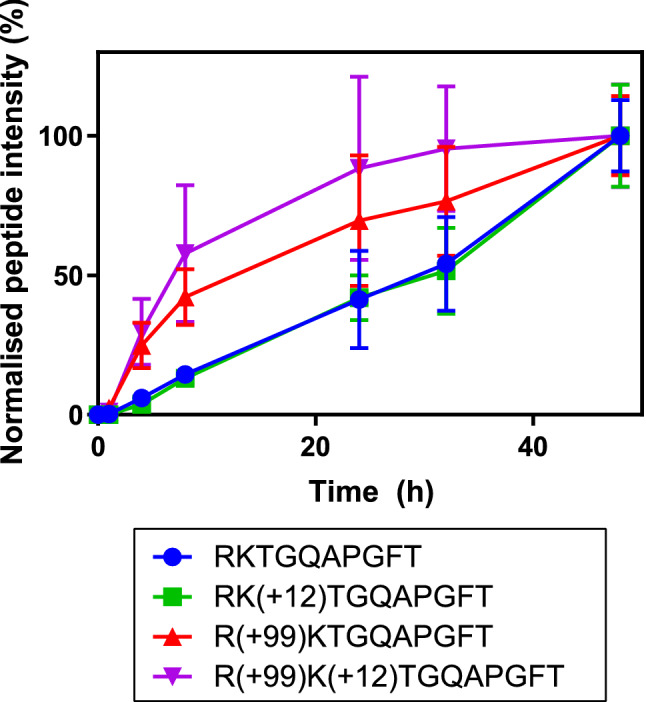
Normalised data of RKTGQAPGFT related peptides in **CYT-FG128-1** subjected to cathepsin S degradation. Peptide areas were first corrected for the internal standard peptide angiotensin-III. Peptides containing Δm + 99 (formaldehyde-glycine) adducts show a faster formation than peptides without such adducts. Error bars represent SD of three LC–MS runs.

Faster cleavage at GLFG↓RKTGQAPGFT is necessary to get faster formation of GLFG. Faster formation of the R(+ 99)KTGQAPGFT peptide compared to the unmodified peptide would indicate that formation of the Δm + 99 modification contributed to the faster formation of GLFG in formaldehyde/glycine-treated samples. The intensities of the modified RKTGQAPGFT peptides during the various timepoints were normalised to compare the kinetic profile of these peptides (Fig. 5). Peptides that contained a Δm + 99 adduct (*i.e.*, 2 formaldehyde groups and 1 glycine group) or Δm + 111 adduct (99 + 12) were formed much faster than the native peptide or the peptide with the + 12 adduct, the last two showing nearly identical formation kinetics. This further supports the finding that glycine adducts on R38 enhance proteolytic cleavage by cathepsin S, whereas modifications that do not involve glycine near this cleavage site (e.g.*,* at K39) do not.

### Enzymatic degradation of a modified synthetic peptide

**Figure 6:**
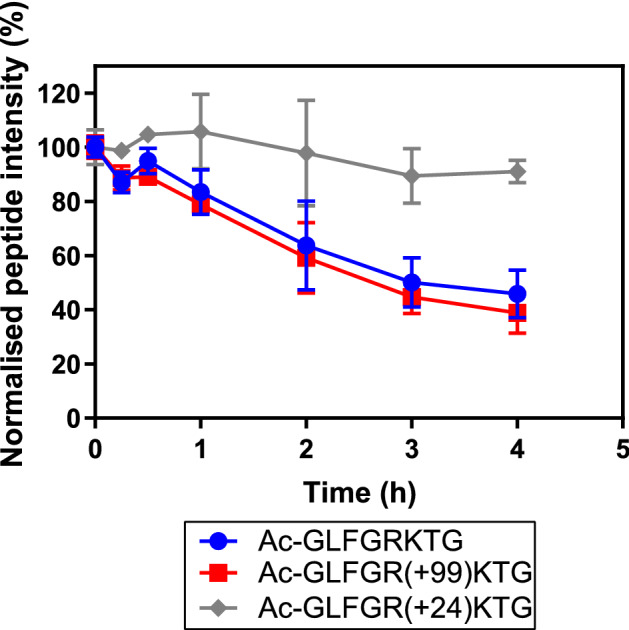
LC–MS peak area of the synthetic peptide Ac-GLFGRKTG and its modified analogues as function of cathepsin S digestion time. Peak areas are corrected by using an internal standard. The native peptide and its Δm + 99 analogue show similar kinetics, whereas the crosslinked Δm + 24 peptide shows a much slower degradation speed. Error bars represent SD of a triplicate of three peptide solutions.

The increased formation of the GLFG peptide upon digestion by cathepsin S of cytochrome c could have two reasons: local unfolding of the protein could make the cleavage site more accessible to the enzyme, or the enzyme could have a preference for the new, modified, arginine side chain. To rule out the latter, a synthetic peptide, Ac-GLFGRKTG, was subjected to formaldehyde/glycine treatment. The mixture of resulting peptides was further purified by SPE to remove free formaldehyde and glycine, and subsequently subjected to enzymatic degradation. Peptides containing a crosslink between the lysine and arginine (i.e., a Δm + 24 adduct) were much more resistant to proteolytic degradation, despite the cleavage site being at the N terminal side of the arginine. Peptides containing the Δm + 99 adduct were not degraded substantially faster than the native peptide. These results strongly suggest that the observed increase in GLFG formation in the formaldehyde/glycine-treated cytochrome c is due to changes in protein folding (discussed below) induced by the chemical modifications rather than by the adduct formation per se (Fig. 6).

### Changes in secondary structure and thermostability of formaldehyde-treated cytochrome c

**Figure 7:**
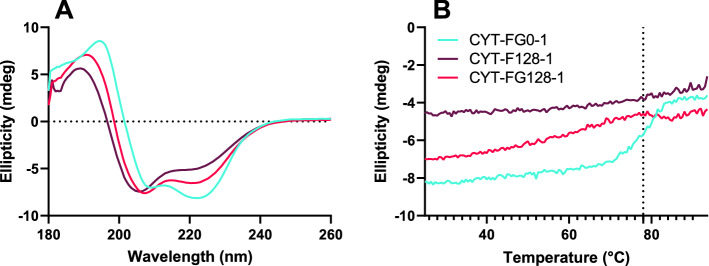
Circular dichroism spectroscopy analysis of **CYT-FG0-1**, **CYT-F128-1** and **CYT-FG128-1**. (**A**) Averaged (n = 3) and smoothed (5 points) CD spectra of **CYT-FG0-1**, **CYT-F128-1** and **CYT-FG128-1** in 20 mM phosphate buffer, pH 7.4. (**B**) CD analysis of protein unfolding temperature (unsmoothed) measured at 220 nm.

To be able to compare conformational differences between untreated and formaldehyde-/glycine-treated cytochrome c, CD spectra of **CYT-FG0-1**, **CYT-F128-1**, and **CYT-FG128-1** were measured and the ratio between the 208 and 222 nm signals was determined. **CYT-FG128-1** and **CYT-F128-1** resulted in different CD spectra compared both to **CYT-FG0-1** and to each other, indicating treatment-induced changes in secondary structure (Fig. 7). To compare the various samples, the ratio between the CD signals at 208 and 222 nm was determined. For **CYT-FG0-1** this ratio was 0.85, **CYT-FG128-1** and **CYT-F128-1** had ratios of 1.16 and 1.41, respectively. The unfolding temperature was determined by measuring CD at 220 nm. **CYT-FG0-1** unfolded near 78 °C while both **CYT-F128-1**, **CYT-FG128-1** did not unfold below 94 °C, indicating that these samples were more thermostable than native cytochrome c. In general, studies that correlate protein thermostability to stability towards proteolytic enzymes have shown that the more thermostable a protein is, the slower it is degraded by proteases.^24,25^ However, these studies mostly use native proteins or proteins with natural amino acid mutations, whereas the structural changes caused by formaldehyde treatment could alter the conformation to make it more accessible to proteases; this treatment can also rigidify this altered conformation, which could lead to enhanced thermostability.

Contrary to previous publications^17^ and our expectations, the incubation of model proteins with glycine and formaldehyde enhances the protein’s degradation speed upon exposure to cathepsin S. Cathepsin S has two important advantages over the previously used trypsin. Firstly, the lysine and arginine residues are among the most affected amino acids upon formaldehyde treatment, which are the exact amino acid residues after which trypsin cleaves. It is very likely that modified lysine and arginine residues are not readily recognised by trypsin anymore. Secondly, the cathepsin S used in our study is a more biologically relevant protease in the context of antigen processing. Moreover, because of its preference for hydrophobic-branched amino acids in the P2, P1′ and P3′ position of the substrate, it does not have the same limitation regarding altered cleavage sites as trypsin does.^26^ These hydrophobic-branched amino acids are not affected by formaldehyde, so only the effect of other neighbouring amino acids (primary structure) and overall protein folding (secondary and tertiary structure) contribute to the degradation kinetics.

Overall, subjecting proteins to formaldehyde or formaldehyde and glycine results in changes in proteolysis rates, leading to an enhanced degradation speed. For specific formaldehyde-glycine adducts we have shown that it is not due to changes in primary structure, but most likely due to changes in protein conformation. This effect is not limited to formaldehyde-glycine adducts, as it was also observed when the proteins were treated with only formaldehyde. Furthermore, using a model peptide, we have shown that formaldehyde-induced crosslinks increase protease resistance. This shows that while some modifications increase proteolytic resistance, the majority of the modifications actually enhance the susceptibility of the protein towards enzymatic proteolysis. Admittedly, the effects seen with the model proteins chosen in this study may not hold true for every protein. However, given the structural diversity of the model proteins used (e.g., size, function, lysine content, disulphide bonds, proteolytic resistance), it is expected that many other proteins will behave similarly. This could have consequences for the immunogenicity and thus the efficacy of vaccine products, since endo-lysosomal degradation results in in situ formation of T-cell epitopes that provide induction of T-helper cells and cytotoxic T-cells.^27^ With regard to vaccine production the chemical modifications caused by the detoxification processes, used for, e.g., diphtheria and tetanus toxins or poliovirus vaccines, could alter antigen processing. This emphasises the importance of ensuring good batch to batch consistency, as variation in the detoxification process might result in variation of the efficacy of the final vaccine product. As variations in the detoxification process can alter the protease resistance of antigens, assays that measure the stability of a vaccine to enzymatic proteolysis have the potential to confirm a consistent detoxification process and in turn contribute to the reduction or replacement of animal tests when combined with other in vitro assays.

## Experimental procedures

### Preparation of formaldehyde and glycine treated proteins

Three model proteins were treated with formaldehyde and glycine, essentially as described by Metz et al.^14^ Bovine serum albumin (BSA, Serva), Bovine β-lactoglobulin A (Sigma-Aldrich) and equine cytochrome c (Sigma-Aldrich) were dissolved in phosphate buffered saline (PBS, pH 7.2, Gibco) to obtain 1.5 mg/mL stock solutions. To 700 µL of these stock solutions per reaction condition, glycine (1 M solution in PBS) and formaldehyde (1 M solution in PBS) were added to achieve 0, 2, 16, 80 or 128 mM formaldehyde and glycine in the final solution. Additional PBS was then added to obtain a total volume of 1,400 µL. Samples were placed in an oven at 37 °C for 6 weeks. A buffer exchange to sodium citrate (100 mM pH 6.0) was then carried out by using Amicon 3-kDa spinfilters (Merck). Protein concentrations were determined by A280 measurements on a SynergyMX using a Take3 microplate (BioTek). Samples were stored at − 20 °C until further treatment.

### Preparation of cytochrome c treated with isotopically labelled formaldehyde and glycine

Cytochrome c was dissolved in PBS to obtain a 1.25-mg/mL stock solution. Formaldehyde and deuterated formaldehyde solutions were diluted in PBS to obtain 1 M solutions. Glycine and glycine-1-^13^C were dissolved in water to obtain 1 M solutions. To aliquots of the cytochrome c solution, either unlabelled formaldehyde and glycine, D_2_-formaldehyde and glycine-1-^13^C, unlabelled formaldehyde, or D_2_-formaldehyde solutions were added to achieve 1 mg/mL cytochrome c with 128 mM of the additives. A control sample was prepared with addition of PBS. All samples were then placed in an oven at 37 °C for 7 days. A buffer exchange to sodium citrate (100 mM pH 6.0) was then carried out by using Amicon 3-kDa spinfilters (Merck). Protein concentrations were determined by measuring A280 on a SynergyMX using a Take3 microplate (Biotek).

### Enzymatic degradation of proteins

Protein samples (Table 2) were subjected to proteolytic degradation by *E. coli*-derived recombinant human cathepsin S (Merck). Reactions were carried out in triplicate, in 200 µL total volume containing 100 mM sodium citrate buffer (pH 6.0), 2 mM ethylenediaminetetraacetic acid (EDTA) and 2 mM dithiothreitol (DTT), with 40 µg protein and 20 ng cathepsins S. Samples were placed in an oven at 37 °C. For the 6-week incubation samples, an aliquot of 10 µL was transferred to 90 µL PBS containing 1 µM E-64 (Sigma-Aldrich) as cysteine protease inhibitor for subsequent SEC analysis upon reaching the desired time point. For all samples, an aliquot of 5 µL was transferred to 95 µL water containing 5 vol% DMSO, 0.1 vol% formic acid and 1 fmol/µL angiotensin-I (Sigma-Aldrich), angiotensin-III (Sigma-Aldrich) and oxytocin (Sigma-Aldrich) for LC–MS analysis. Samples for SEC analysis were stored at 4 °C; LC–MS samples were stored at − 20 °C.

**Table 2:** Reaction conditions of protein formaldehyde treatment.

Reaction condition code	Protein	Formaldehyde concentration (mM)	Glycine concentration (mM)	Incubation time (weeks)
BSA-FG0-6	Bovine serum albumin	0	0	6
BSA-FG2-6	Bovine serum albumin	2	2	6
BSA-FG16-6	Bovine serum albumin	16	16	6
BSA-FG80-6	Bovine serum albumin	80	80	6
BSA-FG128-6	Bovine serum albumin	128	128	6
BLG-FG0-6	ß-lactoglobulin A	0	0	6
BLG-FG2-6	ß-lactoglobulin A	2	2	6
BLG-FG16-6	ß-lactoglobulin A	16	16	6
BLG-FG80-6	ß-lactoglobulin A	80	80	6
BLG-FG128-6	ß-lactoglobulin A	128	128	6
CYT-FG0-6	Cytochrome c	0	0	6
CYT-FG2-6	Cytochrome c	2	2	6
CYT-FG16-6	Cytochrome c	16	16	6
CYT-FG80-6	Cytochrome c	80	80	6
CYT-FG128-6	Cytochrome c	128	128	6
CYT-FG0-1	Cytochrome c	0	0	1
CYT-F128-1	Cytochrome c	128^a^	0	1
CYT-FG128-1	Cytochrome c	128^a^	128^b^	1

^a^The reaction was performed with 128 mM CH_2_O and 128 mM CD_2_O separately, and the reaction products were mixed 1:1 based on protein concentration afterwards.

^b^The reaction was performed with 128 mM glycine and 128 mM glycine-1-^13^C separately, and the reaction products were mixed 1:1 based on protein concentration afterwards. CD_2_O and glycine-1-^13^C were used in the same reaction.

### Preparation and enzymatic degradation of formaldehyde-modified synthetic peptide Ac-GLFGRKTG

Ten mg of the synthetic peptide Ac-GLFGRKTG (Pepscan) was dissolved in water (1 mL). Of this solution, 100 µL was added to 125 µL glycine solution (1 M), followed by 665 µL water, 100 µL PBS (10X Cal Biochem) and 1 µL formaldehyde solution (37 wt% 12.3 M). This mixture was placed in an oven at 37 °C for 24 h. Excess formaldehyde was removed by using a C18 solid phase extraction (SPE) cartridge (Waters) with an ASPEC GX-271 automated SPE system (Gilson). The sample was loaded slowly (50 µL/min), washed with water containing 0.1 vol% formic acid (200 µL/min, 600 µL) and eluted with 60 vol% acetonitrile in water and 0.1 vol% formic acid (100 µL/min, 600 µL). This fraction was then collected and dried in a vacuum centrifuge (Eppendorf Concentrator plus) and reconstituted in 1 mL water to obtain an approximate 1-mg/mL mixture of Ac-GLFGRKTG and its modified analogues. The products were checked by LC–MS and were in agreement with previously published modifications.^15^ Subsequently, 4 µL (4 µg peptide equivalent) of the peptide mixture was diluted in sodium citrate buffer (pH 6.0 100 mM) containing DTT (2 mM) and EDTA (2 mM) to a total volume of 200 µL. Then, cathepsin S (0.69 µL, 1 µg) was added and the sample was placed in an oven at 37 °C for 4 h. This was performed in triplicate. At various time points (0 h, 15 min, 30 min, 1 h, 2 h, 3 h, 4 h), 1-µL aliquots were taken and diluted in 999 µL water containing 5 vol% DMSO, 0.1 vol% formic acid and 1 fmol/µL angiotensin-I, angiotensin-III and oxytocin and stored at − 80 °C until LC–MS analysis.

### LC–MS analysis

The partially digested proteins were analysed by reversed phase nanoscale LC–MS on an Orbitrap Fusion Lumos Tribrid mass spectrometer (Thermo Fisher), essentially as described by Meiring et al.^28^ For each analysis, 10 µL sample was injected and loaded onto the trapping column (Reprosil-Pur C18-AQ 5 μm, Dr. Maish, Germany; 20 mm long × 100 μm inner diameter). The trapping column was subsequently washed with 100% solvent A (0.1 vol% formic acid in water) for 10 min at a flow rate of 5 µL/min. After trapping, the flow was switched to the analytical column (Reprosil-Pur C18-AQ 3 μm, Dr. Maish, Germany; 25.6 cm long × 50 μm inner diameter) with a flow rate of 100 nL/min. A gradient with increasing solvent B (0.1 vol% formic acid in acetonitrile) was started from 8 vol% solvent B to 34 vol% B in 30 min, followed by a steeper gradient to 58 vol% in 5 min. The column was then washed with 85% B for 5 min and equilibrated for the succeeding analysis with 100 vol% A for 10 min. Using nanoelectrospray ionisation (ESI) the column was coupled to the Orbitrap Fusion Lumos Tribrid. Data Dependent Acquisition (DDA) was used, with MS^1^ scans measured in the 300–1500 m/z mass range, in the orbitrap at 120,000 FWHM resolution with a cycle time of 2 s, a maximum injection time of 100 ms and automatic gain control (AGC) target of 2 × 10^5^. Fragmentation was achieved by using both collision-induced dissociation (CID) and electron transfer dissociation (ETD). The isolation window was set to 1.6 Da with an offset of 0.25 Da. The CID normalised collision energy was 35% with the activation Q set to 0.250. CID was used for abundant (> 1 × 10^6^) precursor ions with a charge state of 1, and precursor ions with charge states > 1 and an intensity of at least 5 × 0^5^. ETD reaction times were based on the calibration settings. ETD was used for abundant peptides (> 1 × 10^6^) with charge states ≥ 2. MS^2^ scans were measured in the orbitrap at 15,000 FWHM resolution with an AGC target of 1 × 10^5^, employing CID and ETD (100 ms and 200 ms injection times, respectively).

### MS data analysis

The raw data was analysed by using PEAKS 8.5 (Bioinformatics Solutions Inc.) using the DENOVO, PEAKSDB, PTM and SPIDER and Label-Free Quantification (LFQ) modules. Known formaldehyde-glycine adducts were included as variable modifications in the DENOVO and PEAKSDB search.^14,15^ The error tolerances were set to 3 ppm parent mass error tolerance and 0.01 Da fragment mass error tolerance The resulting peptide areas were corrected for the angiotensin-III internal standard. The isotopically labelled digestion samples **CYT-F128-1** and **CYT-FG128-1** after 48 h of digestion were analysed with MsXelerator (MsMetrix) to obtain a list of isotopic doublets of peptides that should contain formaldehyde or formaldehyde-glycine adducts.^21^ The 48 h samples were then measured again with a targeted analysis. Subsequently, another PEAKS search was performed to identify these modified products. MS spectra that PEAKS could not identify were manually interpreted. Accessibility was calculated by using GETAREA on 1HRC.^29^ The location of α-helices, β-sheets and turns within cytochrome c were calculated by using STRIDE on 1HRC.^30^

### SEC

Degradation kinetics were followed by determining the maximum peak height of the intact protein with SEC using an ACQUITY UPLC 125 Å 1.7 µm 4.6 mm × 150 mm, 1 K-80 K Protein BEH SEC Column (Waters) and a Waters H-Class ACQUITY UPLC, with UV detection at 205 nm and column oven at 40 °C. The mobile phase was 100 mM sodium phosphate buffer at pH 6.8 for BSA and β-lactoglobulin, and 50 mM phosphate buffer with 450 mM NaCl at pH 8 for cytochrome c. The injection volume for all samples was 5 µL. The flow rate was 0.3 mL/min.

### Circular dichroism (CD)

Prior to CD analysis, a buffer exchange through dialysis (3-kDa, Thermo Fisher) on **CYT-FG0-1**, **CYT-F128-1** and **CYT-FG128-1** was performed to achieve 20 mM phosphate buffer, pH 7.4. CD spectra were acquired on a Chirascan Circular Dichroism Spectrometer (Applied Photophysics). CD spectra were measured from 260 to 180 nm with steps of 0.5 nm. Spectra were measured in triplicate, averaged and smoothed over 5 points. For temperature ramping experiments performed in 100 mM sodium citrate buffer, pH 6.0, CD was measured at 220 nm and the temperature was increased 1 °C/min from 25 °C up to 94.5 °C.

## Supplementary information

Supplementary data S1

Supplementary data S2

Supplementary data S3

Supplementary data S4

Supplementary data S5

Supplementary data S6

Supplementary data S7

Supplementary data S8

Supplementary data S9

Supplementary data S10

Supplementary data S11

Supplementary data S12

Supplementary data S13

Supplementary data S14

Supplementary information
