# Supplementary material for: Formaldehyde treatment of proteins enhances proteolytic degradation by the endo-lysosomal protease cathepsin S
Source: Sci Rep. 2020 Jul 14;10:11535. doi: 10.1038/s41598-020-68248-z (PMC7360561; doi:10.1038/s41598-020-68248-z)
Supplement: Supplementary file 15 — Supplementary information [file 41598_2020_68248_MOESM15_ESM.docx]

**Formaldehyde treatment of proteins enhances proteolytic degradation by the endo-lysosomal protease cathepsin S**

**Thomas J. M. Michiels^1,2^, Hugo D. Meiring^2^, Wim Jiskoot^1^, Gideon F. A. Kersten^1,2^, Bernard Metz^2*^**

^1^Division of BioTherapeutics, Leiden Academic Centre for Drug Research (LACDR), Leiden University, Leiden, 2333 CC, the Netherlands
^2^Intravacc, Institute for Translational Vaccinology, Bilthoven, 3721 MA, the Netherlands
*[Bernard.metz@intravacc.nl](mailto:Bernard.metz@intravacc.nl)

Contents:

- Supplementary Figure S1
- Supplementary Figure S2
- Supplementary Figure S3


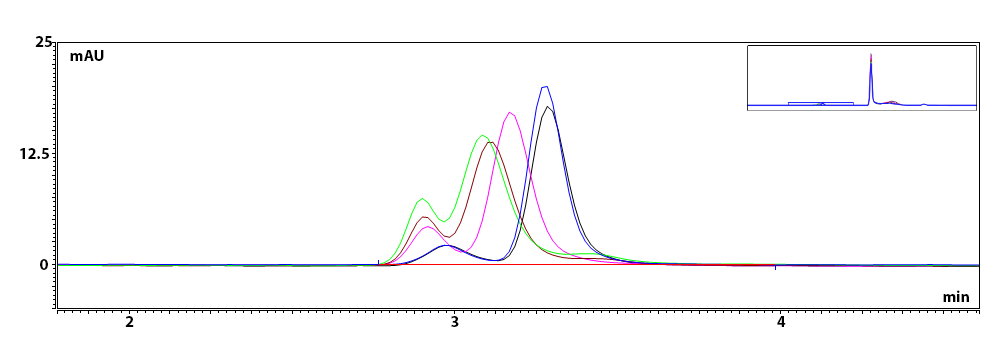


**Supplementary Figure S1**. SEC chromatogram of undigested products BSA-FG0-6 (blue), BSA-FG2-6 (black), BSA-FG16-6 (magenta), BSA-FG80-6 (brown), BSA-FG128-6 (green). See table 2 for explanation of sample codes.


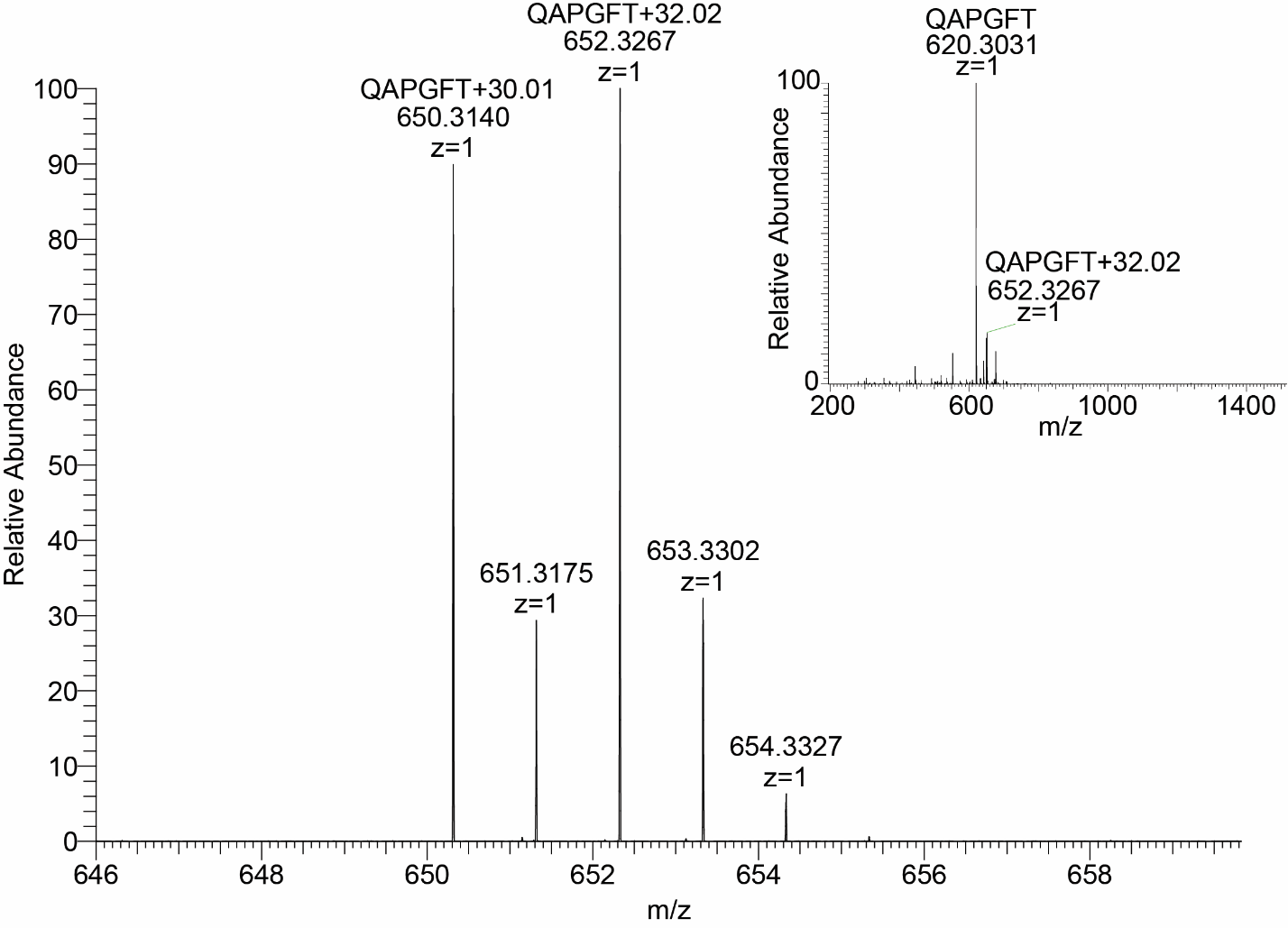


**Supplementary Figure S2.** Average MS1 spectrum of a representative spectral doublet caused by the addition of 1 formaldehyde (CH_2_O or CD_2_O) molecule to peptide QAPGFT.


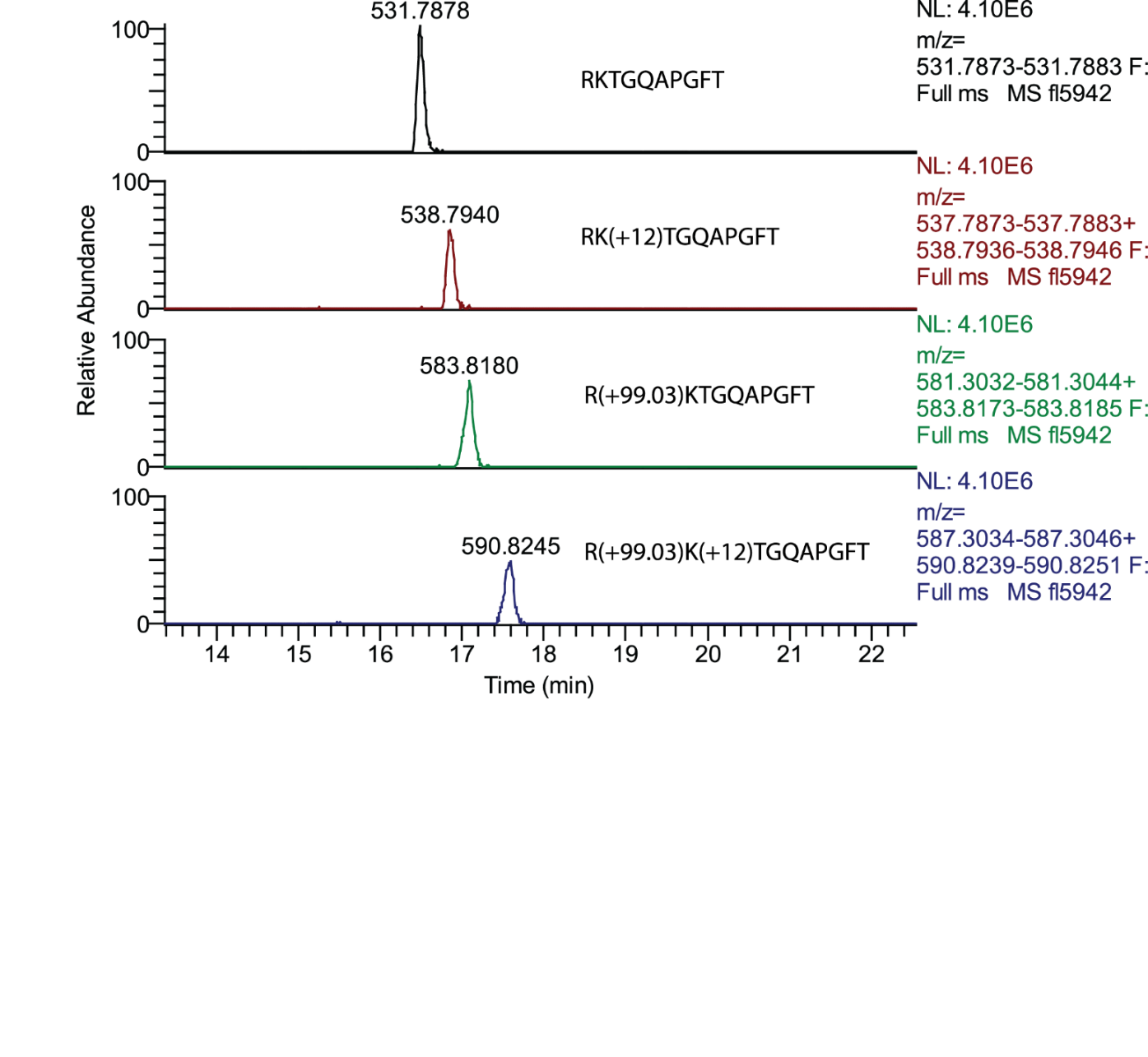


**Supplementary Figure S3**. Extracted Ion Chromatogram of modified RTGQAPGFT peptides from cytochrome C treated with 128 mM formaldehyde and glycine (1:1 heavy:light) for 1 week, after 48 hour digestion with cathepsin S.
